# Intersectoral Ward Rounds on Patients Admitted to Temporary Twenty-Four-Hour Accommodations in Denmark: Case Study

**DOI:** 10.5334/ijic.5688

**Published:** 2022-02-11

**Authors:** Julie Grew, Maj Thomsen, Michaela Louise Schiøtz

**Affiliations:** 1 Center for Clinical Research and Prevention, DK; 2 North Zealand Hospital, DK

**Keywords:** audits, ward round team, intersectoral, temporary accommodation, municipal care, elderly medical patients

## Abstract

**Introduction:**

Temporary twenty-four-hour accommodations (TTAs) are municipal beds for elderly patients discharged from the hospital with acute treatment, care and/or rehabilitation needs that cannot be met in their own homes. TTAs are staffed by nurses and nursing assistants who are not authorized to prescribe or modify medications. At North Zealand Hospital one third of the many readmissions from a TTA within eight days after discharge have been assessed as preventable.

**Description:**

A hospital-based team rounded on 268 patients at TTAs from May 2017 to October 2019 to promote integrated care. This study aimed to assess the efficacy of the rounding by auditing patient cases. A physician, a nurse, and a pharmacist from the hospital; a general practitioner; and one or two TTA nurses audited 17 cases.

**Discussion:**

Obtaining access to all electronic patient records and reconstructing information shared across sectors were not feasible in all cases.

**Conclusion:**

- An overview of the course of treatment was provided in most cases
- The patient’s health was enhanced in most cases and to a considerable or determining degree in half of cases
- Medication was optimized in most cases
- The succeeding course of treatment was enhanced in more than half of the cases
- Readmission was prevented in some cases.

## Introduction

As is the case in many other countries, the growing population of elders in Denmark has prevalent complex health problems, such as multiple concomitant chronic diseases, and is expected to further strain the healthcare system [1]. In Denmark, elderly patients often receive treatment, rehabilitation, or both in hospital, municipality and general practice settings, requiring communication and coordination among healthcare professionals across institutions and sectors. However, the organization of the Danish healthcare system is broadly recognized as a structural barrier to intersectoral cooperation and communication [2,3]. The healthcare system is organized into five regions and 98 municipalities. Regions manage hospitals and are responsible for disease treatment, whereas municipalities are responsible for health promotion, prevention and rehabilitation [2]. General practice provides treatment, prevention and rehabilitation and serves as a gatekeeper for regional and municipal healthcare services; it is a separate healthcare sector because general practitioners (GPs) are self-employed [3].

Due to the division of responsibilities between sectors and accelerated care pathways, many elderly patients who are discharged from the hospital return to their home municipality with substantial treatment, care and rehabilitation needs. Municipalities in the Capital Region of Denmark have established temporary twenty-four-hour accommodations (TTAs), providing a certain number of beds for individuals discharged from the hospital with acute treatment, care and/or rehabilitation needs that cannot be met in their own homes with home care or home nursing support. TTAs are staffed by nurses and nursing assistants who are not authorized to prescribe or modify treatments or medications. A physician is required to start, change or stop medical treatment or address concerns about an individual, and TTA staff contacts the patient’s GP or, less frequently, the hospital.

The three sectors—regions, municipalities and general practice—use separate information systems with limited information-sharing capabilities. TTA staff must rely on hospital staff to share necessary information about individuals residing in TTAs. (However, healthcare professionals across sectors have online access to information about medication and vaccines for all patients via the Common Medicine Card, an electronic system covering all Danish citizens.)

Frequent readmissions—defined as hospitalizations occurring within a well-defined period after a previous discharge [4]—among elderly patients and frequent medication errors in the Danish healthcare system have been documented [5,6]. Siloed sectors with separate responsibilities and IT systems and limited cross-sectoral communication have been identified as plausible causes for readmissions and medication errors [2,7].

At North Zealand Hospital and the municipalities in the hospital’s coverage area in the Capital Region of Denmark, a substantial number of patients aged >65 years have been readmitted [5]. In 2015, an audit was conducted of 45 medical records belonging to citizens who had been readmitted within eight days after discharge from North Zealand Hospital to a TTA in three of the eight municipalities in the hospital’s coverage area. The review panel judged one third of the readmissions as preventable and recommended that, among other changes, increased physician attendance at TTAs could improve integration of care by increasing coherence of care pathways that cross sectors. Integrated care is defined here as coherent care pathways across healthcare sectors.

In 2016, a three-year quality improvement project began with the aim of promoting integrated care for elderly medical patients who were discharged from the hospital to municipal TTAs in the hospital’s coverage area. The project was developed and conducted collaboratively by North Zealand Hospital, eight municipalities (Alleroed, Fredensborg, Frederikssund, Gribskov, Halsnaes, Helsingoer, Hilleroed and Hoersholm), and general practice in the same area. The project included four tracks:

- Triage—developing, testing and implementing a tool for systematic assessment and prioritization of patients at TTAs in participating municipalities
- Faster sampling—procuring C-reactive protein testing equipment, calibrating and standardizing equipment, and transporting microbiological samples from TTAs to central labs
- Good discharge— developing a check list for hospital nursing staff to ensure TTA staff receives relevant information and a collaborative model for pharmacist dispensing and packaging of medications before patients bring them to TTAs
- Intersectoral ward rounds—rounding by a team of healthcare professionals on selected patients at TTAs in the eight municipalities from North Zealand Hospital.

The aim of this study is to assess the efficacy of the intersectoral ward round model by auditing patient cases.

## Ethical approval

According to Danish legislation [8], projects classified as quality care development do not need approval from the Danish Data Protection Agency. The North Zealand Hospital management and the management in the eight municipalities have approved data collection and data sharing in the project, which has been performed in accordance with the General Data Protection Regulation rules of conduct. Informed consent to participate in the project was obtained from patients at their first encounter with the ward round team.

## The model for intersectoral ward rounds

The aim was to develop a model that could promote integrated care through hospital physician rounding on selected patients in TTAs. Planned indicators of success included:

- Improvement of patients’ health status to a degree that would be impossible or difficult in existing health services
- Reduction in inappropriate readmissions, i.e., preventable returns to the hospital due to conditions that can be treated in municipalities
- Increased coordination of treatment across sectors
- Perceptions by patients that they are receiving the help they need*
- Increased quality of care without increased economic costs, i.e., despite immediate increased resource use, overall costs are unchanged as integrated care reduces patients’ needs for treatment and rehabilitation*.

*The two latter bullets were evaluated by other research teams using patient interviews [9] and register data [10], respectively.

At the outset of the project, the ward round team consisted of a medical specialist in general medicine, a nurse, a pharmacist, and a biomedical laboratory technician. As the project proceeded, it became evident that the competences of the latter two team members were better used at the hospital, where the rounding team could also reach them in case of questions. The pharmacist and lab technician also played a central role in developing the good discharge and faster sampling tracks. In addition, a TTA nurse and/or nursing assistant who was familiar with the patient participated in the rounding. When possible, the patient’s next of kin and general practitioner were also included. In the last part of the project, a model in which the hospital nurse was replaced by a young physician was tested. The patients whose cases were included in this report stayed in TTAs during the period in which the ward round team consisted of a medical specialist in general medicine and a nurse.

Througout the project patients were included in ward rounds in different ways; the criteria, though, remained unchanged. The majority of the time, including the period in which the cases for the audit was selected, the patients were included in the following two ways: The physician and the nurse contacted all TTAs, which had a collective total of 240 beds, each morning and selected patients most likely to benefit from intersectoral rounding on the basis of information provided by TTA nursing staff. In addition, TTA staff were encouraged to contact the ward round team if feedback, advice or assistance from a hospital physician was needed. The criteria for inclusion were that patients were aged 65+ years and had been discharged from North Zealand Hospital. In addition, inclusion was based on an individual dialogue between the physian and the TTA nurses aiming to identify patients with a medical need that could benefit from a comprehensive assessment from a hospital-based physician with the possibility to prevent a readmission. Included patients were typically newly discharged after long or repeated hospital admissions where most of the recent health information was primarily available in the hospitals’ electronic records.

After obtaining informed consent from the patient to participate in the project, the team conducted ward rounding consisting of:

- Collecting and reviewing patient information from hospital and municipality electronic patient records
- Completing a thorough physical examination of the patient, frequently complemented by blood and/or microbiological tests and assessment of cognitive and functional status
- Reviewing and, as needed, adjusting medications
- If relevant and in collaboration with the patient and his/her next of kin, preparing a plan for future treatment
- Preparing and distributing a summary to the patient’s GP and TTA staff
- Following up as determined by the patient’s needs.

Between May 2017 and October 2019, the team conducted ward rounds on 268 patients.

## Evaluation

### Case selection

Patient cases were retrospectively selected for auditing in reverse chronological order from among closed cases on a randomly selected date. The most recently closed patient cases were selected until two cases from each of the eight participating municipalities were identified. Three patient cases from Hilleroed Municipality were selected because one case had been used in an earlier pilot test. An assumption of this method is that any early issues related to team composition or activities had already been identified and resolved. Selected patient cases are considered representative of all patients on whom the team rounded.

### Audit process

Audits took place in September-October 2019. Participants in each audit included a medical specialist, a nurse and a pharmacist from North Zealand Hospital, a general practitioner from the hospital’s coverage area and one or two nurses (or, in one case, a nursing assistant) from the TTA where the patient was staying at the time of the ward round. No participants were involved in any of the audited patient cases.

Audits were based on the hospitalization before the patient’s first contact with the ward round team (index hospitalization). Audit material consisted of information each participant could find from electronic records at the hospital and municipality. GPs lack access to electronic records from both locations and received audit material on the day of the audit that included an overview of the care pathway, notes from the index admission, the hospitalization discharge summary and records from the ward round team.

**Figure 1:**
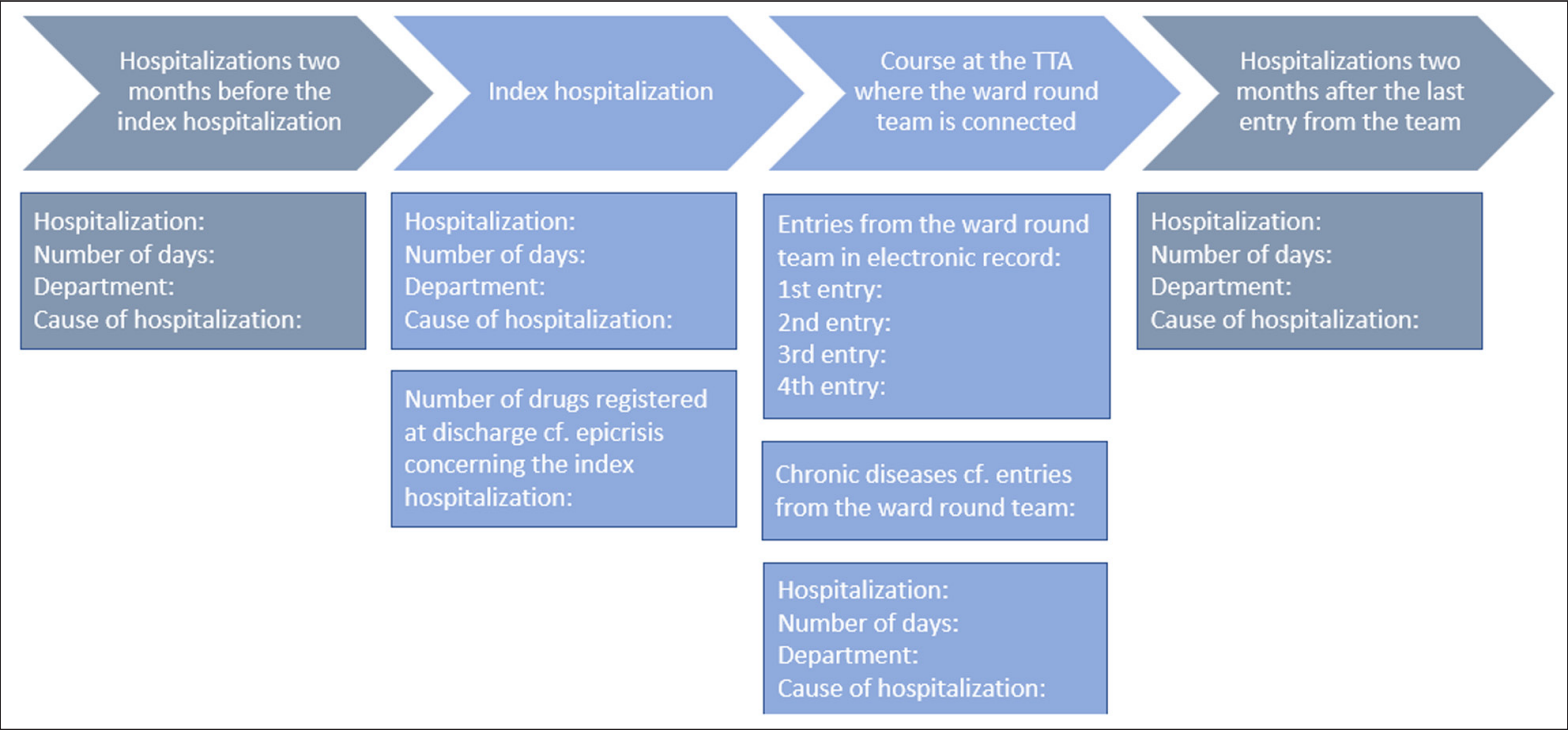
Overview of events included in audits.

***Figure 1*** depicts an outline generated for each case before audit. It gave participants an overview of the index hospitalization, any hospitalizations two months before and two months after the index hospitalization, and the ward round process at the TTA.

Audits focused on:

1. The index hospitalization, including information shared between municipality, general practice and hospital, assessment and treatment, and medications
2. The subsequent care pathway at the TTA, including when and why the ward round team was contacted
3. Ward round team interventions, including medication changes and readmission prevention
4. Changes in the patient’s health status.

The audit form was developed in collaboration between the project manager from North Zealand Hospital and the two evaluators. It comprised questions that participants from the hospital and the municipality were asked to answer before audits began and others that all participants were asked to answer together during audits. Some questions pertained to facts about the cases and others solicited a professional valuation of the quality of the care pathway and the efforts of involved healthcare professionals based on the available material (***Table 1***).

**Table 1:** The audit form questions.


QUESTIONS TO BE ANSWERED *BEFORE* THE DAY OF THE AUDIT BY HOSPITAL-BASED PHYSICIANS, NURSES AND PHARMACISTS REGARDING THE HOSPITALIZATION IMMEDIATELY BEFORE THE PATIENT’S FIRST CONTACT WITH THE WARD ROUND TEAM

Was a referral sent by the physician under whose order the patient was hospitalized? If yes, describe the quality of the referral. **(only to be answered by physicians and pharmacists)**

Was a manually updated admission report sent from the municipality? If yes, when was the report received at the hospital? Describe the quality of the admission report.

Which diagnoses, symptoms and problems were addressed during the hospitalization?

What is your assessment of the investigation conducted during the hospitalization? Could the patient have benefitted from further investigation during the hospitalization? **(only to be answered by physicians)**

What is your assessment of the treatment initiated during the hospitalization? Could the patient profitably have been treated further or differently during the hospitalization? **(only to be answered by physicians)**

In your judgment, was it relevant to hospitalize the patient? **(only to be answered by physicians)**

In your judgment, were there any inappropriate patient medications before and during the hospitalization? **(only to be answered by physicians and pharmacists)**

Were any medication changes made during the hospitalization? **(only to be answered by physicians and pharmacists)**

In your judgment, was the patient appropriately medicated by the time of discharge? **(only to be answered by physicians and pharmacists)**

In your judgment, was there any risk of impending readmission by the time of discharge?

If yes, could anything have been done to reduce the risk of readmission?

What information was sent electronically from the hospital to general practice and the municipality? Was any information delivered only in paper form? In your judgment, was the information adequate for the general practitioner and municipality to handle further care?

Which conditions of the patient’s situation made it relevant to refer the patient to a TTA?

**QUESTIONS TO BE ANSWERED *BEFORE* THE DAY OF THE AUDIT BY THE NURSES AT THE TTAS REGARDING THE CARE PATHWAY AT THE TTA**

When did the patient stay at the TTA (times and dates for arrival and departure)?

What information was received in the municipality from the hospital by the time of the patient’s arrival at the TTA? In your judgment, was the information adequate for the municipality to handle further care?

Was the nursing assessment prepared and, if yes, when? Was the information from the hospital considered in the nursing assessment?

Which vital parameters were assessed at the reception of the patient at the TTA? Could other parameters profitably have been assessed?

How much time passed from discharge until the ward round team attended to the patient for the first time?

Why did the staff at the TTA contact the ward round team?

How often were vital parameters measured before the ward round team was contacted? Could other parameters profitably have been measured or with another frequency?

In your judgment, could the staff at the TTA have prevented deterioration of the patient’s health status before the ward round team attended to the patient?

In your judgment, could any contact with a general practitioner at an earlier stage have prevented deterioration of the patient’s health status?

Have any changes in medication been made or have any concerns about inappropriate medication been documented during the patient’s stay at the TTA?

**QUESTIONS TO BE ANSWERED JOINTLY *DURING* THE AUDIT REGARDING THE EFFORT OF THE WARD ROUND TEAM**

Describe the content of the effort of the ward round team

In your judgment, did the effort of the ward round team improve the patient’s state of health?

In your judgment, did the effort of the ward round team prevent an admission within eight days following the patient’s last contact with the team?

In your judgment, did the ward round team contribute to creating an overview of the patient’s immediate situation? In your judgment, did the effort of the ward round team improve the succeeding care pathway?

In your judgment, did the effort of the ward round team optimize the patient’s medication?

Are there any other elements of the care pathway that the effort of the ward round team affected?

In your judgment, did it make a difference that the ward round team had easy access to information in the hospital’s electronic patient records?

Could parts of the effort of the ward round team profitably have been performed during the hospitalization?

Is it likely that a general practitioner could have made a similar effort?

Overall assessment of the effort of the ward round team?

**QUESTIONS TO BE ANSWERED JOINTLY *DURING* THE AUDIT REGARDING THE TOTAL CARE PATHWAY ACROSS SECTORS**

Which elements of the care pathway worked particularly well across sectors?

What were the challenges in the care pathway across sectors?

In an ideal healthcare system, what could have been done better?


The audits were audio recorded, and the answers to the questions were registered in a paper version of the audit form for each case by the first author. After each audit the first author listened to the recording to confirm and supplement the written notes. For those questions soliciting a professional valuation of the quality of the care pathway, any disagreements among the participants were documented. In all cases the audit panel shared each others’ opinions on the quality of the care pathway.

The written material was analyzed using counting as for questions with predefined possible answers. Answers to open-ended questions and clarifying answers to closed-ended questions were coded thematically and then categorized in a simple content analysis [11].

## Results

### Characteristics of audited cases

The 17 patients whose care was audited ranged in age from 66 to 93 years, with an average age of 81 years. They were diagnosed with multiple chronic conditions, had multiple health problems and were treated with multiple drugs. In many cases, they were hospitalized due to several ambiguous symptoms appearing simultaneously. Symptoms most frequently observed among the patients at index hospital admission were increased infection rates, pain, confusion, urinary tract infection, nausea and vomiting, dehydration, increased blood sugar levels, diarrhoea, fever, oedema and ulcers. Index hospitalizations lasted from a few hours to 87 days. Eleven patients were hospitalized one or more times in the two months before the index hospitalization, and 11 patients were hospitalized during or immediately after the ward round team intervened. Nine patients had passed away between ward round interventions and audits: four patients died immediately or shortly after the ward round intervention and five patients died 6–12 months later.

### Characteristics of index hospitalizations

The review panel assessed examinations and treatments performed during index hospitalizations as appropriate in most cases. Yet, in more than half of audited cases, reviewers identified a risk of readmission in the near future, most frequently due to an increased general risk of infection, continuing pain or infection, or because treatment was assessed as incomplete or ineffective. In half of the cases, reviewers assessed that hospital staff could have increased their efforts to reduce the risk of readmission by, for example, extending the index hospitalization or conducting further investigation.

Across the 17 audited patient cases, a range of difficulties were identified related to both communication between hospital, municipality and general practice and medications.

#### Difficulties related to hospital admission

- In half of audited cases, the physician who requested the admission sent a referral but the information in the referral was assessed as adequate in only a third of these cases
- Missing information most frequently included a medication list and information about tentative diagnoses and secondary diagnoses
- In less than half of cases, a manually updated admission report was sent from the municipality to the hospital (mandatory when the patient has received care from the municipality before the hospitalization) and, in five of seven cases in which the report was sent, it was sent the day after admission or later
- In half of the cases, some medications listed at admission were assessed as inappropriate by the review panel.

#### Difficulties related to hospital discharge

- In all cases, a discharge summary was sent to general practice, but the information in the summary was assessed as inadequate in more than half of the cases
- Missing information most frequently included a medication list, test results, the reason for the index hospitalization and a treatment plan
- In more than half of cases, information such as a discharge report, a care plan and a rehabilitation plan were sent to the TTA, but the information was assessed as adequate in only one case
- In half of the cases, some medications listed at discharge were assessed as inappropriate.

### The TTA care pathway

In audited cases, patients stayed in TTAs a mean of 37 days (range, 6–100). Most patients stayed for less than a month, whereas a small group of patients stayed for two months or more. On average, two days (range, 1-5) passed between hospital discharge to the first visit from the ward round team, with the exception of a single case in which 66 days elapsed. The most frequent reasons TTA staff contacted the ward round team were lack of information about the care pathway before the patient’s arrival at the TTA, uncertainty or concern about the patient’s health status, lack of a plan for future care, need for review and professional discussion, and continuing symptoms after discharge, typically pain.

In more than half of cases, the nursing assessment was completely or partially prepared at the TTA and, in most cases, vital parameters (body temperature, pulse, blood pressure, respiration and level of consciousness) were measured when patients arrived at the TTA. From that point until the first visit from the ward round team, vital parameters were measured systematically in slightly less than half of the cases. In more than half of the cases, the review panel judged that vital parameters should have been measured more often or that other measures should have been included. In most cases, the panel assessed that neither TTA staff nor earlier contact with general practice could have prevented deterioration of the patient’s health before the ward round team was contacted.

### The intervention of the ward round team

The review panel members were asked to describe the most important elements of the ward round team intervention in the 17 patient cases. The elements highlighted most frequently were:

- A comprehensive assessment of the patient and the care pathway
- Medication review and adjustment
- Planning for further care
- Discussing the patient and sharing information with the TTA staff.

The ward round team ameliorated some of the difficulties that were identified in the previous course of treatment. In addition, several elements were identified as essential for the ward round team:

- Involving the patient and his/her next-of-kin
- Access to all information in the hospital’s IT system
- Contact with medical specialists in the hospital and GPs.

The review panel was asked to assess the overall effect of the ward round team in terms of defined indicators:

- An overview of the course of treatment was provided in most cases
- The patient’s health was enhanced in most cases and to a considerable or determining degree in half of cases
- Medication was optimized in most cases
- The succeeding course of treatment was enhanced in more than half of the cases
- Readmission was prevented in some cases.

In most cases, the effort of the ward round team was assessed as impossible or difficult to accomplish for healthcare professionals in existing health services, primarily because of the time required, visit frequency, continuity of care, and rapid response to TTA staff. The review panel assessed that, in half of the cases, a GP could not have made a similar effort because it would require more time or more frequent visits than a GP can usually offer.

## Discussion

The results must be interpreted with caution; in some audited cases, obtaining access to all the electronic patient records relevant for the care pathway was not feasible. In other cases, it was not possible to reconstruct with certainty the care pathway and information shared between hospital, municipality and general practice. Nevertheless, we believe that the results reflect issues related to intersectoral care in the Danish healthcare system and demonstrate a clear impact of the ward round team on care pathways.

Multidisciplinary teams are common elements in integrated care models for older people, particularly in Europe and North America, but also in Asia and Oceania [12]. For people aged 65+ years multidisciplinary teams are used in hospital settings to perform comprehensive geriatric assessments (CGA) [13], to optimise health outcomes in acute care [14], and to improve transition from emergency departments to community [15] and in primary care settings to optimize medication prescribing [16]. The effort of the ward round team share many similarities with CGA, which is defined as “a multidimensional, interdisciplinary diagnostic process focused on determining the medical, psychological, and functional capabilities of a frail elderly person to develop a coordinated and integrated plan for treatment and long-term follow-up” [17]. Several studies from the US report on CGA provided during hospitalization at specialized geriatric wards [18–22] and CGA taking place during hospitalization and continuing after discharge [23,24]. Some studies deal specifically with CGA including outpatient follow-up provided by a hospital-based mobile team of health professionals [25,26]. Other initiatives resembling the ward round team, that is, multidisciplinary teams performing comprehensive assessments of elderly people after hospital discharge have been tested, among other places, in Australia, where elderly patients were offered CGA and a multidisciplinary intervention after discharge from the emergency department to home [27] and in the US, where high risk patients were offered an integrated care transition program including home visits by a multidisciplinary team after discharge [28]. More recent research on CGA includes multidisciplinary teams visiting elderly people in community settings, e.g. in Sweden, where CGA was compared with usual outpatient care [29]. National examples are post-discharge home visits by a GP and a municipal nurse [30], post-discharge home visits by a hospital-based geriatric team [31], and early geriatric follow-up visits to nursing home residents [32]. At Odense University Hospital, a team of physicians and nurses conduct ward rounds at a community rehabilitation center and, at Vejle Hospital, physicians conduct ward rounds at a TTA in Vejle Municipality [33]. No published evaluations are available for the latter two projects. At Aarhus University Hospital, CGA for elderly patients referred to a community rehabilitation unit has been compared with standard care [34].

In the UK, during the last twenty years a variety of intermediate care services has been launched to bridge the gap between hospital care and community care. Provided in patients’ homes, in community-based settings or in discrete facilities in acute hospitals some of the aims are to reduce preventable hospital admissions and to promote independent living [35,36]. Similar initiatives have been established in our neighboring country Norway, the health care system of which shares numerous characteristics with the Danish one. Here, elderly and chronically ill patients can be discharged to a temporary stay at an intermediate care hospital to improve coordination of care and follow-up [37]. Additionally, 24-hour municipal acute units have been established in all Norwegian municipalities to avoid hospitalization or readmission. Eligible patients are sent to a unit before or instead of hospitalization [38,39].

A realist review of integrated care programs concluded that facilitators for the success of integrated care programs are multidisciplinary team relationships based on trust; providers that commit to and understand the model; support from leaders; and successful implementation [40]. A scoping review of implementation of integrated care for older people identified core components to be care continuity and transitions; enabling policies and governance; shared values and goals; person-centred care; multi-/inter-disciplinary services; effective communication; case management; and needs assessments for care and discharge planning. Specific barriers and facilitators include, among other things, funding, organizational leadership, structure of existing services, and culture/philosophy of the system; as well as intervention size and complexity, resources and credibility [41]. The present evaluation points to resources as the primary barrier for the success of the interprofessional ward round team: the main part of the work was done by a high-wage professional and was time-consuming. An economic analysis estimated that the project produced additional expenses within a three-months follow-up period [10]. All in all, multidisciplinary teams and CGA have been shown to be important elements of integrated care programs for the elderly just as several other elements of the interdisciplinary ward rounding, that is, care management and effective communication between health professionals [41]. Results are inconclusive regarding effects on integrated hospital discharge for frail elderly people specifically [42] and due to lack of power and systematism findings from this study are only indicative of a positive effect of interdisciplinary ward rounding on hospital-to-community transition. Randomized controlled trials with sufficient numbers of participants are necessary if we are to find evidence of any connection between interdisciplinary ward rounding for frail elderly people and integration of care.

The ward round team was discontinued when the project ended. Instead, North Zealand Hospital, the municipalities and general practice are collaborating to promote integrated care for elderly medical patients with multiple chronic conditions and complex health problems. Some elements accomplished by the ward round team could be completed during hospitalizations, while other elements could be accomplished by GPs. The competencies required are available in the hospital, TTAs and general practice. However, organizational changes are required to allow healthcare professionals to take the time required for new and existing tasks, including flawless exchange of robust information across sectors.

The review panel made several suggestions for managing the challenges observed after the project ended and the ward round team ceased operation. Hospital physicians expressed a desire to extend the length of hospitalizations to allow time for thorough examinations and follow-up on observations. Review panel participants suggested facilitating communication by telephone, e.g. by establishing a hotline to GPs and hospital physicians that bypasses telephone hours and receptionists. To improve medication management, the panel suggested that GPs be required to update medication lists in the event of acute hospitalizations (not a current requirement) and that each patient be asked about medications at hospital admission, arrival at a TTA and in general practice. Finally, review participants suggested that a GP or a hospital physician be permanently affiliated with every TTA, replacing the ward round team to some degree.

## Lessons learned

Essential characteristics of the ward round team distinguish it from other actors in healthcare services:

- *Time for a continuous and systematic effort* comprising frequent visits to thoroughly review the care pathway and medications and involve the patient and his/her next-of-kin, for example, in planning further assessment and treatment
- *Access to all information* in hospital electronic patient records on site at TTAs
- *Readily accessible to TTA staff* during weekdays; quick responses when needed.

Nevertheless, the process of identifying eligible patients and ward rounding itself were time-consuming, not least because of the distances the ward round team travelled between the hospital and various TTAs.

## Conclusion

Elderly patients with multiple chronic conditions and complex health problems benefitted from a thorough review, a treatment plan, and coordination of care. For these patients, it is crucial to create an overview of their care trajectory and provide coordinated care. However, in the existing healthcare system, these needs are often not adequately addressed.

Before the ward round intervention, prerequisites for integrated care were often not present. The communication channels did not always work well or were not used optimally, and essential information was not shared across sectors or shared information was often inadequate. This resulted in missing information at the hospital, municipalities and general practice, challenges with appropriate use of medications, and ambiguity about diagnosis, treatment, medication and further care. These widespread issues were addressed by the ward round team, resulting in an overall increase in care quality and integration in audited cases according to the audit panel’s assessment.

Further research on how to promote integration of care in the transition from hospital to home is needed. Future studies should address ways to overcome the difficulties related to communication across hospital, municipality and general practice at hospitalization and discharge and should include effect evaluation. Research on barriers and facilitators of adequate information would be a valuable precursor of intervention studies.

A new initiative leaving treatment responsibility for frail elderly people to the department of discharge until 72 hours after discharge has been decided to start in 2022 in all hospitals in the Capital Region of Denmark. This will provide TTA staff and home nurses with a direct phone line to a physician or a nurse who knows the patient. Evaluation of effects on readmissions and communication at discharge (quantity and quality of information from the hospital) and assessment of patient and staff experiences is highly recommended.
